# Comparing ability and norm-referenced scores as clinical trial outcomes for neurodevelopmental disabilities: a simulation study

**DOI:** 10.1186/s11689-022-09474-6

**Published:** 2023-01-17

**Authors:** Cristan Farmer, Audrey Thurm, Jesse D. Troy, Aaron J. Kaat

**Affiliations:** 1 Neurodevelopmental and Behavioral Phenotyping Service, National Institute of Mental Health, Bethesda, MD USA; 2 Department of Biostatistics and Bioinformatics, Duke University School of Medicine, Durham, NC USA; 3 Feinberg School of Medicine, Northwestern University, Chicago, IL USA

**Keywords:** Ability score, Growth scale value, Rasch analysis, Item response theory, Neurodevelopmental disability, Rare genetic condition, Floor effect, Clinical trials, Clinical outcome assessment, Endpoints

## Abstract

**Background:**

For genetic conditions associated with neurodevelopmental disorder (GCAND), developmental domains such as motor ability, thinking and learning, social abilities, and communication are potential intervention targets. Performance on measures of developmental concepts can be expressed using several types of scores. Norm-referenced scores are intended for the diagnostic context, allowing for the identification of impairment relative to age-based expectations, and can exhibit dramatic floor effects when used in individuals with more significant limitations. Person ability scores, which are derived via Rasch analysis or item response theory, are available on many standardized tests and are intended to measure within-person change. However, they have not been used or evaluated as primary endpoints in GCAND clinical trials. In this study, we simulated a series of parallel-arm clinical trials under several chronological age and impairment conditions, to compare empirically the power and type I error rate of operationalizing test performance using ability scores rather than norm-referenced scores.

**Results:**

Using the Vineland Adaptive Behavior Scales as the example, we demonstrated an advantage in statistical power of ability scores over norm-referenced scores at extreme levels of impairment. This advantage was at least partially driven by floor effects in norm-referenced scores. For simulated conditions where impairment was less severe, ability scores outperformed norm-referenced scores, but they were more similar. The type I error rate closely approximated the nominal type I error rate of 5% for both scores.

**Conclusion:**

The results of this simulation demonstrate a substantial power and interpretative advantage of ability scores over norm-referenced scores for studies of GCAND that will enroll participants with high levels of impairment. These results are expected to generalize to studies of developmental concepts, regardless of the etiology or specific test. However, the relative advantage of ability scores is expected to be even greater for tests with a higher floor than the Vineland.

**Supplementary Information:**

The online version contains supplementary material available at 10.1186/s11689-022-09474-6.

## Background

Neurodevelopmental disorders such as intellectual disability are prominent in many rare genetic conditions affecting children [1]. In fact, a genetic diagnosis is now considered likely for most of the people with severe or profound intellectual disability [2]. For these genetic conditions associated with neurodevelopmental disorder (GCAND), developmental domains such as motor ability, thinking and learning, social abilities, and communication are potential intervention targets [3]. The recent momentum in GCAND pharmaceutical clinical trials (e.g., Fragile X syndrome [4];) and gene or genomic therapy (e.g., Angelman syndrome and Rett syndrome [5, 6]) has spurred interest in the psychometric profiles of measures intended for developmental concepts.

Developmental concepts are expected to change over time in some predictable way, and so developmental measures are often norm-referenced. Norm-referenced scores (which may be referred to as “standard” or “scaled” scores) are distribution-based derivations, meaning that they are an empirically based estimate of the proportion of the proband’s peer population expected to achieve the same score or lower. Norm-referencing makes it possible to contextualize the performance of a child against that of their peers, which is essential to the diagnostic context wherein disability is defined relative to age-based expectations. However, because norm-referenced scores do not reflect absolute performance, interpretation of change on the normative scale is difficult. Change in a normative score does not necessarily imply change in performance; deterioration, stability, and even absolute improvement but at a slower rate than same-aged peers can result in worsening of norm-referenced scores. This indeterminacy is especially concerning for neurodegenerative conditions, which GCAND often are. Norm-referenced scores are also known to exhibit poor reliability at extreme values (e.g., [7]), where “extreme” refers to scores more than 2 SD away from the average of the individual’s normative age group. Given that this is a cutoff for many diagnoses, this poor reliability is consequential in the study of neurodevelopmental disability. Further, because many tests do not offer norm-referenced scores beyond 3–4 SD below the mean, floor effects significantly reduce their usefulness for individuals with GCAND [8, 9]. Floor effects become more prominent as people with GCAND fall further behind their age-peers, making this limitation of norm referenced scores particularly salient for older samples.

These limitations of norm-referenced scores have prompted the search for alternative clinical trial endpoints (e.g., [10]), including the use of other available score types [11]. Many measures of developmental concepts offer a suite of scoring options that are complementary to the norm-referenced scores, including raw scores, age equivalents, and person ability scores. The psychometric profiles of these scores differ in important ways. Between-subject variability might be expected to be lower for norm-referenced scores and age equivalents, and higher for raw scores and person ability scores. While norm-referenced scores are measured at the interval level, raw scores and age equivalent are ordinal, which introduces the need for larger sample size and difficulty in interpretation of change when used as clinical trial endpoints. Person ability scores, however, were designed to measure within-person change on an interval level. Although they have not been widely used as clinical trial endpoints, ability scores have existed for as long as test developers have used Rasch modeling and item response theory (e.g., [12]). These models assume that an individual’s score on a particular item depends on both the person’s ability and the difficulty of the item (for technical information, see [13–15]). These person and item parameters are conceptualized as latent variables on the same scale, and so by using information from the Rasch or IRT calibration in the development sample, a person’s ability can be estimated based on their test performance.

Test-specific aliases are often given to ability scores, which are available on a variety of developmental tests (e.g., W-scores on the Woodcock-Johnson, Change Sensitive Scores on the Stanford-Binet Intelligence Scale, Growth Scores on the Leiter International Performance Scale, Growth Scale Values on the Peabody Picture Vocabulary Test). Ability scores have been well-established in academics, where measurement of individual change is the explicit goal (e.g., MAP testing RIT scores and Lexile scores). Until recently, ability scores were employed in only a few observational studies of neurodevelopmental disorder [16–19], but several planned and current GCAND trials use ability scores as primary or secondary endpoints (e.g., NCT03952637, NCT05067582). Thus, the need for evidence to support the psychometric profile of ability scores is growing [11].

The goal of this study was to provide empirical support for the assertion that person-ability scores may be statistically superior to norm-referenced scores when used as endpoints in clinical trials for neurodevelopmental disability [9]. Owing to floor effects and increased variability of norm-referenced scores at the extremes of the distribution, we hypothesized that the advantages of ability scores will be more pronounced for samples with severe impairment, which reflect the level of functioning observed in GCAND. We simulated a series of randomized clinical trials to systematically explore whether the use of person ability scores rather than norm-referenced scores as the study endpoint improves power and/or type I error, and whether this depends on the age and impairment level of the participants. We chose adaptive behavior as the exemplar developmental concept, but we note that the study is about ability scores generally rather than adaptive behavior specifically. This information may be used to guide the design and analysis of future clinical trials, as well as outcome measure development, particularly for severe neurodevelopmental disability.

## Results

**Fig. 1:**
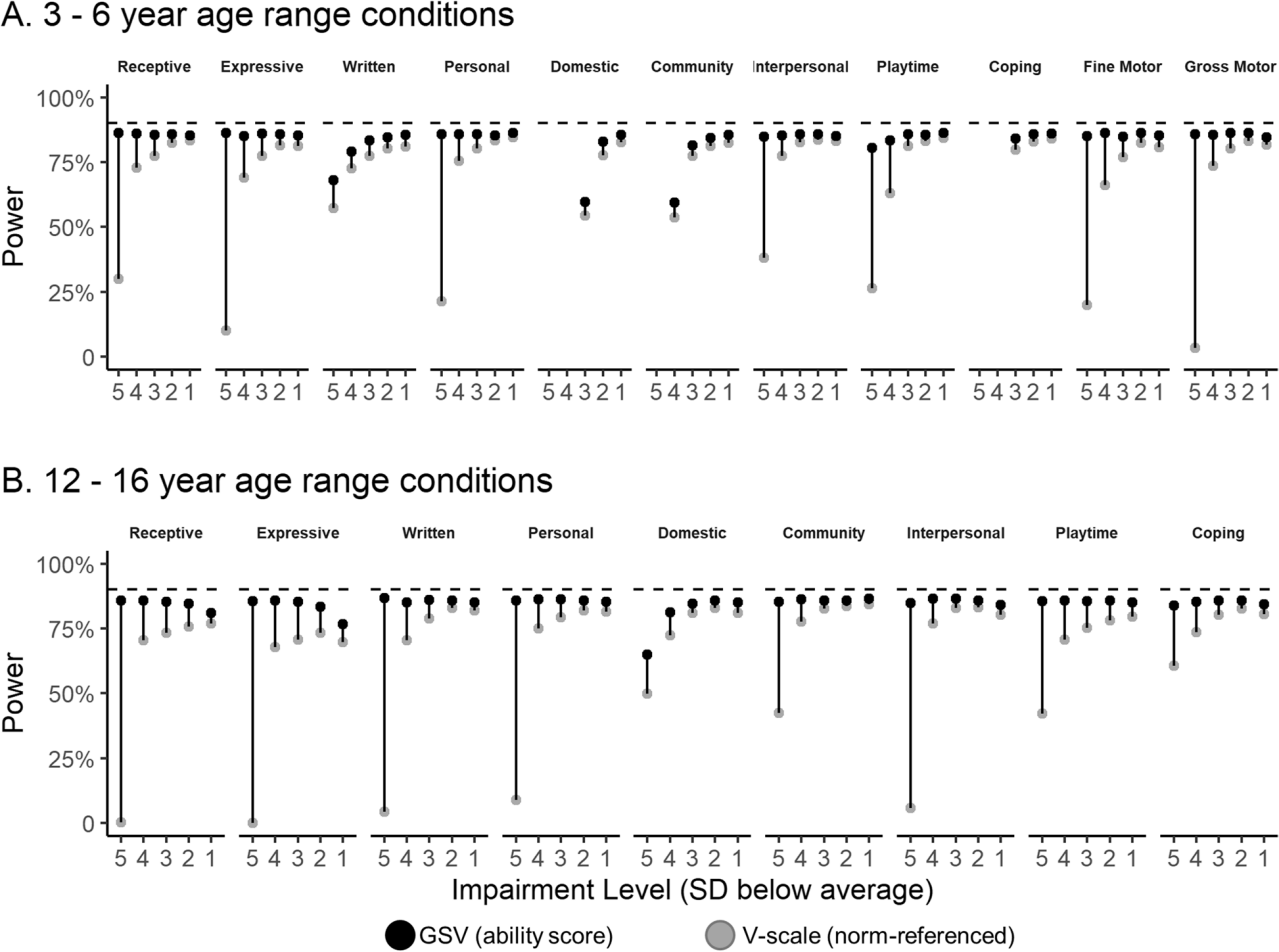
Power when performance is operationalized as GSV versus V-scale. **A** The results for each impairment level in the younger age range conditions. **B** The results for each impairment level in the older age range conditions. Impairment level refers to SD below average, such that 5 corresponds to the most impaired condition. Power is the proportion of 5000 simulations where the *t* test of the group difference controlling for baseline score had a *p* value < .05. The dotted reference line is at 90%

A table with all results for all simulation scenarios is provided in Additional file 1: Supplementary materials. We hypothesized that the advantage of GSV over V-scale would be more pronounced for older and more impaired samples. The median empirical power for studies using GSV ranged 85–86%, while that for V-scale ranged 9–83% (Table 1). Thus, our hypothesis was supported by an advantage of GSV over V-scale that was progressively larger for each degree of impairment in the sample, and slightly more pronounced for the older population than the younger (see Table 1, Fig. 1).

**Table 1:** Observed power for GSV and V-scale, summarized across subdomains. Impairment level refers to SD below average, such that 5 corresponds to the most impaired condition

Age range	Impairment level	Subdomains	GSV power	V-scale power	Decrease in power when using V-scale instead of GSV				
			*Median*	*Median*	*Min*	*Q1*	*Median*	*Q3*	*Max*
3–6 years	5	8	0.85	0.24	0.11	0.52	0.60	0.68	0.83
	4	9	0.85	0.73	0.06	0.08	0.12	0.16	0.21
	3	11	0.86	0.77	0.03	0.04	0.06	0.07	0.09
	2	11	0.86	0.82	0.02	0.03	0.03	0.04	0.05
	1	11	0.85	0.83	0.02	0.02	0.03	0.04	0.05
12–16 years	5	9	0.86	0.09	0.15	0.43	0.77	0.82	0.86
	4	9	0.86	0.72	0.09	0.10	0.12	0.15	0.18
	3	9	0.86	0.79	0.03	0.04	0.07	0.10	0.15
	2	9	0.86	0.83	0.02	0.03	0.03	0.08	0.10
	1	9	0.85	0.80	0.02	0.04	0.04	0.04	0.07

The advantage of GSV over V-scale was most pronounced in the samples with the greatest degree of impairment. It was under these conditions that we also observed complete floor effects, wherein for a given simulated study, *all* participants in the PBO and/or the TRT group received the lowest possible V-scale score (see Additional file 1: Supplementary Table S2). This lack of variability (σ^2^ = 0) meant that the ANCOVA could not be performed. While complete floor effects did not occur at all for GSV, they occurred in 15% (*n* = 14 of 95) of scenarios using V-scale. However, when complete floor effects occurred, they were usually observed in a small minority of simulated datasets. For 8 of the 14 scenarios with complete floor effects, the rate was < 10% (i.e., more than 90% of the 5,000 simulations for the scenario were successful; power for these scenarios ranged from 10.1 to 42.3%). For three other scenarios, the rate of complete floor effects was < 25% (power for these scenarios ranged 3.2 to 8.8%). The three scenarios with high rates of complete floor effects were from the Communication domain in the older age group: 26.2% for Written (power = 4.2%), 67.3% for Receptive (power = 0.18%), and 93.0% of Expressive (power = 0). While complete floor effects were coded as a failure to reject the null hypothesis for the calculation of power, they did not fully explain the very low power of V-scale scores in these scenarios as the rate of failure to reject the null hypothesis outpaced the rate of total floor effects in all cases.

Finally, the type I error rate closely approximated the nominal type I error rate of 5% for both GSV (median [IQR] = 0.050 [0.049, 0.051]) and V-scale (median [IQR] = 0.049 [0.046, 0.050]) across all scenarios. Here too, simulations resulting in complete floor effects were coded as failure to reject the null hypothesis.

## Discussion

Because neurodevelopmental disability is the predominant phenotype for many GCAND, developmental concepts such as motor ability, thinking and learning, social abilities, and communication are potential treatment targets. The most common usage of developmental tests is in a diagnostic context, where the norm-referenced scoring of performance allows for comparison of the individual to age-based expectations. However, the norm-referenced scores are not ideal for a response monitoring context, due to limitations in interpretation, reliability, and responsivity that occur for test takers with extreme impairment. The Rasch modeling or item response theory-based person ability score is intended for measuring within-subject change and may be a superior candidate as a clinical trial endpoint. We observed an overall advantage in statistical power of operationalizing performance as an ability score rather than a norm-referenced score, the magnitude of which was more pronounced as the degree of impairment in the sample increased. We derive two important insights from this pattern of results. First, the more extreme impairment conditions in this simulation are the ones germane to many studies of GCAND, where a significant proportion of the sample will likely be at or near the floor of the norm-referenced scores [20]. Therefore, the results of this study support the use of ability scores rather than norm-referenced scores for many GCAND. Second, in the less impaired conditions, ability scores were still slightly better than norm-referenced scores. Given the limitations in interpretation of norm-referenced scores, the results of this study suggest that even when floor effects are not a concern, ability scores may be preferable endpoints.

In this study, we used simulated performance on the Vineland Adaptive Behavior Scales to evaluate our hypotheses. The results of the study should not be considered specific to the test, however, and should be broadly generalizable to any developmental concept and measure, so long as ability scores have been generated by the test publisher. However, we note a major caveat. The Vineland offers norm-referenced scores up to 5 standard deviations below average (i.e., V-scale = 1), which means that floor effects are less pronounced than for most other developmental tests which have a higher floor (most provide norm-referenced scores only down to 3 or 4 standard deviations below average). Because the advantage of ability score over norm-referenced score on the Vineland is at least partially driven by floor effects, even when they are not complete, the power advantage of ability scores should be more pronounced for other tests with stronger floor effects in norm-referenced scores. Thus, these Vineland simulation results are likely an underestimate of the power advantage of ability scores for other measures.

Further, like other adaptive behavior measures, the Vineland is intended to be applicable to a very wide age range (0–99 years). This means that for most subdomains, both norm-referenced and ability scores can be generated for a participant of any age. This is not true of most other developmental measures, however, which are targeted to a narrower developmental range. For example, the Wechsler family of assessments has unique tests for preschoolers, children, and adults, and norm-referenced scores cannot be obtained for tests which were administered outside of the age range. If a test is at the correct developmental level, however, ability scores can be obtained regardless of the individual’s chronological age. Thus, this simulation would not have been possible with some other tests, where norm-referenced scores corresponding to ability scores might not be available for older participants.

The results of our simulation study must be interpreted in the context of several limitations and additional considerations. Most notably, the results of a simulation study are only generalizable to conditions reflected in the assumptions made by the data generative process. One such assumption is that GSV scores are normally distributed within a given chronological age range. We used the mean GSV, subtest reliability, and the subtest SEM to create a normal distribution from which to draw scores, but by the central limit theorem, even if the population distribution were not normally distributed, we should expect the sample distributions utilized herein to be normally distributed. Parametric tests are built upon the central limit theorem and are robust to its violations, as such the consequences of violating this assumption are small provided a reasonable sample size is employed in practice. Another choice we made in specifying the distribution of GSV scores was to derive an estimated SD, based on the SEM and reliability data for normative age groups. This may have led to either an under- or over-estimated the width of the distribution from which GSVs were drawn, though we have no reason to believe this approach is more likely to bias the result in one direction than in another.

The design of the simulated clinical trials was simple; we chose a between-group comparison at endpoint, controlling for baseline. This reflects a particular approach to the analysis of clinical trials, where it is not the rate of change that is of interest, but rather the degree of change at the primary endpoint. How these results generalize to the former, and to open-label or natural history studies of disorder, is an open question suitable for future study. Based on the results of this study, we expect more precise estimates of rate-of-change in GSV scores than in V-scale scores.

While in this context, we simulated age as factor which should be balanced between groups, a major issue to contend with in a non-randomized study using ability scores is the confounding role of chronological age. For any developmental concept, the rate of change in ability is expected to be a function of chronological age; the desire to account for this fact is, of course, the motivation for norm-referenced scores. However, information from typical development does not export neatly to severe neurodevelopmental disability. Whether, or how closely, the developmental trajectory follows that of normative samples, is an open question with answers that are likely strongly dependent on context (Which disorder? Which level of impairment? Which age range?). This underscores the essential role of high-quality natural history studies of rare genetic conditions, including the potential for such research to serve as external historical controls for open label studies [21]. Thus, the role that person ability scores may play in such research is an area in need of deep consideration and study. We suggest that for tests with a person ability score, these should be reported as part of natural history studies to serve this important role—they are more relevant to the proposed purpose of assessment.

Finally, it is important to note that this simulation study is focused purely on the statistical detection of a given treatment effect and does not inform the question of what type of change might be considered “clinically significant.” The assessment of change in ability scores at an individual level is done by contextualizing the observed change with the SEM [22], or measurement error, but it is not clear that this distribution-based approach is sufficient for clinical trials. As with any endpoint, a significant amount of qualitative and quantitative evidence is needed to identify the amount of change in ability score which is clinically meaningful, and this will vary across concepts and conditions. Several investigators are pursuing this issue (e.g., [23]), and the results of their work will be important context for the current results. Namely, because we used a standardized effect size approach to determine both the effect size and the sample size of the simulated studies, the resulting conditions may be over- or under-powered relative to what the true clinically important effect size.

## Conclusion

Developmental outcomes are, by definition, a moving target, and the host of resulting measurement issues is compounded by the fact that many of our most rigorously developed tools are inappropriate for the most severely affected individuals. Here, we demonstrate that a parallel-group clinical trial may be more successful in accurately detecting an effect of treatment on the development of people with severe disability when using ability scores than with norm-referenced scores. Person ability scores are not a panacea, but this work supports the assertion that they may be a useful tool in the kit of the clinical researcher.

## Methods

### Vineland Adaptive Behavior Scales, Third Edition

The U.S. Food and Drug Administration considers functioning to be amongst the most essential clinical outcome assessments (which include patient-reported outcomes, clinician-reported outcomes, observer-reported outcomes, and performance outcomes) to track, regardless of condition [24], so we focus on adaptive functioning as the exemplar developmental concept. Adaptive functioning is an established part of neurodevelopmental assessment, and it appears in many longitudinal and treatment studies of genetic conditions. The most recent version of the widely used Vineland Adaptive Behavior Scales [25] is the first edition to contain person ability scores, which are called Growth Scale Values (GSV). The Vineland Comprehensive Interview Form is a semi-structured parent/caregiver interview designed to assess adaptive behavior across the lifespan. Items are arranged into 11 subdomains, each belonging to one of four domains (Communication, Socialization, Daily Living, and Motor).

#### Norm-referenced scores

Age-based norms are provided, based on samples constructed to reflect the USA population (per 2014 Census data) in each age range [25]. At the subdomain level, the norm-referenced scores are called V-scale scores and have a population mean of 15 and SD of 3, with a minimum score of 1. Because the Vineland is intended for use in neurodevelopmental disability, the range of norm-referenced scores is wider than for other tests (e.g., the floor of most IQ tests is no more than 4 SD below average). V-scale scores were derived through inferential norming of the raw scores.

#### Person-ability scores

Growth Scale Values (GSVs) are also available at the subdomain level. The Vineland-3 was calibrated using the Andrich rating scale model [26]. The transformation from ability score to GSV used a coefficient of 9.1024 and a subdomain-specific constant (derived using joint maximum likelihood) to achieve a minimum GSV of 10 [26]; the maximum ranges from 110 to 197 depending on the subdomain. They are obtained via lookup table, corresponding to raw subdomain scores, which is publicly available (https://www.pearsonassessments.com/content/dam/school/global/clinical/us/assets/vineland-3/vineland-3-manual-appendices-b-e.pdf). Although the GSV range appears similar across subdomains, GSVs are a unitless measure and therefore cannot be compared or combined across subdomains. As of this writing, the standard errors of measurement (SEM) for GSV, which range from about 2 to about 9 depending on subdomain and age range, are not included in the manual but are available upon request from the publisher.

### Simulation design

We used R version 4.0.2 [27] to simulate a series of randomized clinical trials, wherein cases were randomly assigned to placebo or active treatment and assessed at baseline and endpoint at 6 months. To create relevant studies, we conducted the simulations at two treatment effect sizes (defined below) and for all subdomains. To explore the hypothesis that the advantages of GSV will be more pronounced for samples with severe impairment than for those with more moderate impairments, we manipulated two sample-level factors: sample age range and sample impairment level. The final list of scenarios is provided in Additional file 1: Supplementary Table S1.

#### Sample-level factors

The sample age range factor had two levels: 3–6 years and 12–16 years. While these ranges appear broad and cover a wide range of development, they were intentionally selected to illustrate age ranges commonly included in neurodevelopmental disability research.

Sample impairment on the GSV scale was conceptualized using the normative (V-scale) scores. The impairment factor had five levels, corresponding to 1, 2, 3, 4, and 5 standard deviations below the population mean on the V-scale (i.e., scores of 12, 9, 6, 3, and 1, respectively). First, the average of each level of the chronological age factor (5 years, 0 months and 14 years, 6 months for the young and old conditions, respectively) was used to select a V-scale lookup table (Vineland Scoring Manual Table B.1). From that table, the average raw score for each subdomain associated with V-scale scores of 12, 9, 6, 3, and 1 were returned. The GSV corresponding to this raw score was obtained from Vineland Scoring Manual Table B.2 and used as the mean of the generating distribution described below in step 1.

#### Study design factors

The best practice in study planning is to power the trial based on the smallest effect size which would be clinically meaningful. Because there are currently no data available from which to determine a clinically meaningful effect on the GSV scale, we instead used an effect size-based approach to determine the sample size in the simulated study. Sixty-six participants are required to achieve 90% power for a moderately large standardized mean difference (Cohen’s *d* = 0.80) with 5% alpha. Given the inclusion of baseline score as a covariate, we reduced the sample size proportionally by 1–ρ^2^ ([28], p. 2924) where ρ was defined as 0.8 (see final paragraph of this section). Thus, the sample size used for simulation was 36% of 66, *N* = 24.

To mirror the sample size determination, the simulated treatment effect size was specified as a function of the SEM of the GSV, which was derived through methods described below. The SEM was converted to a standard deviation, and the effect size in GSV units was calculated as 0.8*SD, or a large standardized mean difference. A second condition, wherein the effect size was 0, was used to evaluate type I error.

Finally, to generate correlated data (two timepoints per person), it was necessary to specify a within-subject correlation parameter. The Vineland manual reports test-retest correlations for V-scale, but not GSV, over a period of 12–35 days. These range from 0.69 to 0.87 for the age ranges and subscales used here. Data available to the authors, from a GCAND natural history study, exhibit within-subject GSV correlations ranging from 0.86 to 0.96 between baseline and 6 months. Given this information, we selected a within-subject correlation of ρ = 0.8 for this simulation.

### Dataset generation

**Fig. 2:**
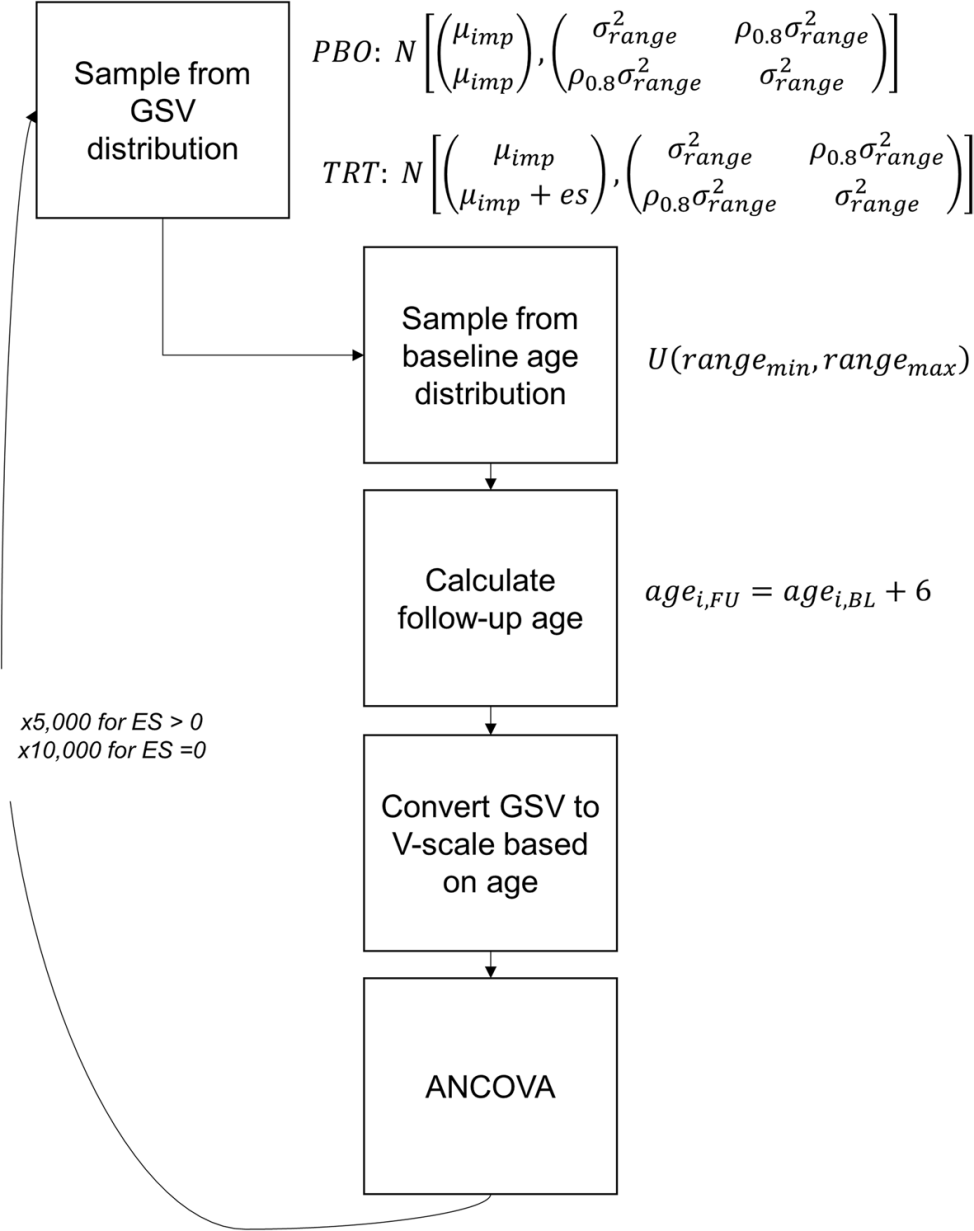
Simulation design. PBO = placebo; TRT = active treatment; ANCOVA = analysis of covariance; GSV = growth scale value (ability score); *range* = age range condition (3–6 years or 12–16 years); *imp* = impairment condition (average V-scale for sample of 1, 2, 3, 4, or 5 SD below average); *es* = effect size condition (zero or large standardized effect). Baseline and follow-up scores were drawn from a bivariate normal distribution, where the within-subject correlation was fixed at *r* = 0.8 and the mean and standard deviation were defined based on the age range and impairment conditions. For the PBO condition, the mean at baseline and follow-up were identical. For the TRT condition, the baseline mean was identical to PBO, but the follow-up mean was shifted by a factor depending on the effect size condition. Baseline age of the simulated participants was drawn from a uniform distribution, and V-scale scores were obtained using a lookup table with age and the simulated GSV score. Finally, the effect of group on follow-up score was calculated based on ANCOVA, controlling for baseline score. This process was repeated 5000 times (large effect size) or 10,000 times (zero effect size) for each combination of conditions

Each scenario was used as the basis for generating 5000 unique datasets (10,000 for the type I error condition). The process for a single dataset is described below and illustrated in Fig. 2.

#### *Step 1*

The placebo group GSV baseline and endpoint scores were drawn from a bivariate normal distribution. The mean of this distribution was determined using the impairment factor described above. The standard deviation of the placebo group GSV score distribution was determined using (a) the chronological age range design factor, (b) the GSV SEM table (Pearson Assessments, Personal Communication, September 30, 2020), and (c) the internal consistency reliability estimates (Vineland Scoring Manual Table 6.1). Both the GSV SEM and reliability estimates are presented by normative age group, such that an age group-specific SD could be calculated using the following formula: $SD=\frac{SEM}{\sqrt{1-r}}$ . Because the chronological age range design factor spanned multiple Vineland normative age groups, we derived the variance of the mixture of *n* age groups (essentially a weighted average that accounts for the dispersion of the means of each age group): ${\sigma}_{mix}^2=\sum_1^n\left({w}_n{\sigma}_n^2\right)+\left(\sum_1^n\left({w}_n{\mu}_n^2\right)-\sum_1^n{\left({w}_n{\mu}_n\right)}^2\right)$, where *w* represents the proportional weight of the age range design factor covered by the Vineland manual age group.

#### *Step 2*

Step 1 was repeated to generate the scores for the active treatment group. These scores were drawn from a bivariate normal distribution, where the mean of the baseline distribution was identical to that of the placebo group and the mean of the endpoint distribution was shifted by adding a constant equal to the treatment effect size (large when the treatment was assumed to be effective, or zero for the simulation of type I error).

#### Step 3

A baseline age vector with length equal to the simulation sample size was drawn with uniform probability and replacement from the range (in months) defined by the chronological age design factor. A follow-up age vector was computed by adding a constant of 6 months to the baseline age vector.

#### Step 4

The dataset resulting from steps 1 to 3 contained randomly generated values for group assignment, baseline age, endpoint age, baseline GSV, and endpoint GSV. The Vineland Manual Table B.2 was used to convert these GSV into raw scores. If multiple raw scores were associated with a single GSV score, the median raw score was selected. The resulting raw scores were then converted into V-scale values based on the age of the case, using Vineland Scoring Manual Table B.1.

### Model of analysis

Each stimulated dataset was analyzed using an analysis of covariance (ANCOVA) model, with baseline score included as a covariate and the effect of group assignment (placebo versus active) the effect of interest. The outcomes of interest in this study were power and type I error. Power is the ability of a test to detect (i.e., produce a p-value below some threshold) a true effect. In this study, power was defined as the proportion of the replications for a given scenario that the two-tailed test of the null hypothesis was rejected at *p* = .05. Type I error is the proportion of tests which incorrectly rejected the null when the true effect size is zero. Given the data generation described above and an alpha of .05, Power should be at 90% and the type I error rate should be near 5%.

## Supplementary Information

**Additional file 1: Supplementary Materials.** Comparing ability and norm-referenced scores as clinical trial outcomes for neurodevelopmental disabilities: A simulation study. **Table S1.** Conditions for each scenario. **Table S2.** Results for each scenario. Power was the primary outcome in this study and is the proportion of simulated studies for each condition which returned a p-value less than .05. Floor effects prevented the calculation of a p-value in some cases. These studies were categorized as failing to reject the null hypothesis (*p*>.05). The median between-group difference for each scenario is also provided for context.

## Data Availability

The code used to generate and process the datasets is provided in the Additional file 1: Supplementary Materials. However, users will need to obtain additional data from Vineland Manual Tables B.1 and B.2, which as of this writing are freely available [29]. These tables are not reproduced here due to copyright. The remaining data that support the findings of this study (SEM values for V-scale and GSV) are available from NCS Pearson but restrictions apply to the availability of these data, which were used under license for the current study, and so are not publicly available. Data are however available from the authors upon reasonable request and with permission of NCS Pearson.

## Abbreviations

ANCOVA: Analysis of covariance
GCAND: Genetic conditions associated with neurodevelopmental disability
GSV: Growth scale value
IQR: Interquartile range
PBO: Placebo
SD: Standard deviation
SEM: Standard error of measurement
TRT: Active treatment
