# Supplementary material for: Comparing ability and norm-referenced scores as clinical trial outcomes for neurodevelopmental disabilities: a simulation study
Source: J Neurodev Disord. 2023 Jan 17;15:4. doi: 10.1186/s11689-022-09474-6 (PMC9843928; doi:10.1186/s11689-022-09474-6)
Supplement: Supplementary file 1 — Additional file 1: Supplementary Materials. Comparing ability and norm-referenced scores as clinical trial outcomes for neurodevelopmental disabilities: A simulation study. Table S1. Conditions for each scenario. Table S2. Results for each scenario. Power was the primary outcome in this study and is the proportion of simulated studies for each condition which returned a p-value less than .05. Floor effects prevented the calculation of a p-value in some cases. These studies were categorized as failing to reject the null hypothesis (p>.05). The median between-group difference for each scenario is also provided for context. [file 11689_2022_9474_MOESM1_ESM.docx]

**Supplementary Materials:** Comparing ability and norm-referenced scores as clinical trial outcomes for neurodevelopmental disabilities: A simulation study

**Corresponding** **Author:** Cristan Farmer, [Cristan.Farmer@NIH.gov](mailto:Cristan.Farmer@NIH.gov)

**Table S1.** See spreadsheet. Conditions for each scenario.

**Table S2.** See spreadsheet. Results for each scenario. Power was the primary outcome in this study and is the proportion of simulated studies for each condition which returned a p-value less than .05. Floor effects prevented the calculation of a p-value in some cases. These studies were categorized as failing to reject the null hypothesis (p>.05). The median between-group difference for each scenario is also provided for context.

**R Code 1:** Obtain blgsv values for Table S1.

**R Code 2:** Calculate standard deviation for mixture of distributions

**R Code 3:** Execute the simulation

**R Code 1: Obtain blgsv values for Table S1**

# the code in this file returns GSVs associated with the average raw score that yields a V-scale score of 1, 3, 6, 9, and 12 for the average age of each group. This GSV is the center of the distribution for baseline scores at a given impairment level for a given age group.

# note that for 7 combinations of age and subscale, the V-scale was not on the lookup table. these combinations were excluded from analysis.

# note that FMO and GMO V-scale scores are not available for the medium and old age groups. these combinations were excluded from analysis.

library(DescTools)

library(tidyverse)

# aes = lookup table for age equivalent based on raw score or GSV

# lookups = lookup table for V-scale score based on age and raw score

# Average age of each group is 60, 114, and 174 months.

# Normative tables for those have MinMonths_GE of 60, 112, and 174.

out.gsv <- function(age, label){ lookups %>%

filter(MinMonths_GE == age,

vscale %in% c(1, 3, 6, 9, 12)) %>% #select relevant rows

mutate(maxscore = case_when(!is.na(maxscore) ~ maxscore,

T ~ minscore),

raw = floor((minscore + maxscore) / 2)) %>% #calculate average raw score for each row

select(subscale, MinMonths_GE, vscale, raw) %>%

left_join(aes, by = c("subscale","raw")) %>%

rename(domains = subscale,

impairmentlevel = vscale,

blgsv = gsv) %>%

select(domains, impairmentlevel, blgsv) %>%

mutate(agelevel = label)}

out <- bind_rows(out.gsv(60, "young"),

out.gsv(112, "medium"),

out.gsv(174, "old")) %>%

mutate(impairmentlevel = as.numeric(impairmentlevel)) %>%

filter(!(agelevel == "medium" & domains %in% c("gmo","fmo")),

!(agelevel == "old" & domains %in% c("gmo","fmo")))

**R Code 2: Calculate standard deviation for mixture of distributions**

library(tidyverse)

# internal_consistency = reliability values from manual, where each row corresponds to standardization subsample and each column corresponds to a subdomain

# gsv_sem = GSV SEM values from publisher, where each row corresponds to standardization subsample and each column corresponds to a subdomain

stats <- full_join(internal_consistency %>%

pivot_longer(cols = rec:fmo, names_to = "subscale", values_to = "r"),

gsv_sem %>%

filter(min_mos >= 36) %>%

pivot_longer(cols = rec:fmo, names_to = "subscale", values_to = "sem"),

by = c("subscale","min_mos","max_mos")) %>%

mutate(ae = ceiling((min_mos + max_mos ) / 2),

sd = sem / sqrt(1 - r),

weight = ((max_mos + 1) - min_mos) / 12,

agemin = case_when(max_mos <= 83 ~ 36,

max_mos <= 143 ~ 84,

T ~ 144))

# AgeEquiv = age equivalent values (ae) corresponding to GSV scores, from manual

medianae <- function(age){AgeEquiv %>%

group_by(subscale) %>%

filter(abs(ae - age) == min(abs(ae - age))) %>%

summarize(medianGSV=floor(median(gsv))) %>%

mutate(ae = as.numeric(paste0(age)))

}

stats<-bind_rows(medianae(unique(stats$ae)[1]),

medianae(unique(stats$ae)[2]),

medianae(unique(stats$ae)[3]),

medianae(unique(stats$ae)[4]),

medianae(unique(stats$ae)[5]),

medianae(unique(stats$ae)[6]),

medianae(unique(stats$ae)[7]),

medianae(unique(stats$ae)[8]),

medianae(unique(stats$ae)[9]),

medianae(unique(stats$ae)[10]),

medianae(unique(stats$ae)[11]),

medianae(unique(stats$ae)[12]),

medianae(unique(stats$ae)[13]),

medianae(unique(stats$ae)[14])) %>%

full_join(stats, by = c("subscale","ae"))

# calculate mixture SD for agegrps covered in study age ranges, including medium age group not used in simulation

# study age ranges are 36 - 83 months, 84 - 143 months, and 144 - 203 months

# this corresponds to agegrp categories (in these tables) 1:6, 7:11, and 12:14

out <- stats %>%

group_by(subscale, agemin) %>%

summarize(sum = sum(weight)) %>%

right_join(stats, by = c("subscale","agemin")) %>%

mutate(p = weight / sum,

term1 = p*sd^2,

term2 = p*medianGSV^2,

term3 = p*medianGSV) %>%

group_by(subscale, agemin) %>%

summarize(term1 = sum(term1),

term2 = sum(term2),

term3 = sum(term3),

avg_r = mean(r)) %>%

mutate(var = term1 + term2 - term3^2,

sd_mix = sqrt(var),

sem_mix = sd_mix*sqrt(1-avg_r)) %>% #use average r value to calculate mixture SEM from mixture SD

filter(!is.na(sd_mix)) %>%

select(subscale, agemin, sd_mix, sem_mix) %>%

ungroup() %>%

rename(domains = subscale)

**R Code 3: Execute the simulation**

# Load necessary datasets

# AgeEquiv = lookup table for age equivalent based on raw score or GSV

# vLooks = lookup table for V-scale score based on age and raw score

# combos = list of scenarios, Table S1. 190 combos for two age groups, 5 impairment levels, 2 effect sizes, and 9 (old age) and 11 (young age) domains, minus impairment level/age combos that do not exist

vLooks$maxscore <- ifelse(is.na(vLooks$maxscore),vLooks$minscore,vLooks$maxscore)

# Load necessary functions

## ---------------------------------------------------------------------------------------------

## rbvn()

## ---------------------------------------------------------------------------------------------

## Generate data from a bivariate normal distribution. Taken from:

## http://blog.revolutionanalytics.com/2016/08/simulating-form-the-bivariate-normal-

## distribution-in-r-1.html

##

## Arguments:

## n - number of observations to generate

## mu1 - mean of 1st component of bivariate normal

## s1 - SD of 1st component of bivariate normal

## mu2 - mean of 2nd component of bivariate normal

## s2 - SD of 2nd component of bivariate normal

## rho - correlation between 1st and 2nd components of the bivariate normal distribution

##

## Return values:

## If all goes well, returns a data frame with correlated data, X1 and X2.

## ---------------------------------------------------------------------------------------------

rbvn<-function (n, mu1, s1, mu2, s2, rho) {

gsv_bl <- rnorm(n, mu1, s1)

gsv_fu <- rnorm(n, mu2 + (s2/s1) * rho * (gsv_bl - mu1), sqrt((1 - rho^2)*s2^2))

as.data.frame( round(cbind(gsv_bl, gsv_fu) ), 0)

}

## ---------------------------------------------------------------------------------------------

## simFX()

## ---------------------------------------------------------------------------------------------

## Simulate a study for a given set of conditions.

##

## Arguments:

## scenario - scenario number in combos file, which refers to a set of 8 conditions

## see first 8 lines of function

##

## Return values:

## Dataframe of length 1 with columns for p-value, significance, parameter estimate, and SE

## for both V and GSV in a study simulated based on the provided conditions

## ---------------------------------------------------------------------------------------------

simFX <- function(scenario){

## Get condition parameters

domains = combos[scenario,]$domains

sampsize = combos[scenario,]$sampsize

corr = combos[scenario,]$corr

agemin = combos[scenario,]$agemin

agemax = combos[scenario,]$agemax

blgsv = combos[scenario,]$blgsv

gsv_true = combos[scenario,]$gsv_true

sd_mix = combos[scenario,]$sd_mix

## Generate data for the control group.

control = rbvn(n=sampsize/2,

mu1=blgsv,

s1=sd_mix,

mu2=blgsv,

s2=sd_mix,

rho=corr)

## Generate data for the treatment group, where treatment effect is present at FU.

treat = rbvn(n=sampsize/2,

mu1=blgsv,

s1=sd_mix,

mu2=blgsv + gsv_true,

s2=sd_mix,

rho=corr)

## Combine the treatment and control groups into a single dataset

control$group = 0

treat$group = 1

study = rbind(treat,control)

## Generate ages of participants at baseline and at 6 months follow-up

study$agebl <- sample(agemin:agemax, size=sampsize, replace=T)

study$agefu <- study$agebl + 6

## Get lookup tables for condition

useAgeEquiv <- AgeEquiv[which(AgeEquiv$subscale == domains),]

useLookups <- vLooks[which(vLooks$subscale == domains),]

## Use lookup tables to convert GSV to V-scale using age

study$gsv_bl_obs <- NA_real_

study$gsv_fu_obs <- NA_real_

study$v_bl <- NA_real_

study$v_fu <- NA_real_

for(i in 1:nrow(study)){

## return observed GSV, which may differ from true GSV if true GSV is not in lookup table

study$gsv_bl_obs[i] <- useAgeEquiv[which.min(abs(useAgeEquiv$gsv - study$gsv_bl[i])),'gsv']

study$gsv_fu_obs[i] <- useAgeEquiv[which.min(abs(useAgeEquiv$gsv - study$gsv_fu[i])),'gsv']

## return raw score for baseline gsv per row

tmpRawBL <- useAgeEquiv[which.min(abs(useAgeEquiv$gsv - study$gsv_bl_obs[i])),'raw']

if(length(tmpRawBL) > 1){

tmpRawBL <- trunc(median(tmpRawBL))

}

## return raw score for fu gsv per row

tmpRawFU <- useAgeEquiv[which.min(abs(useAgeEquiv$gsv - study$gsv_fu_obs[i])),'raw']

if(length(tmpRawFU) > 1){

tmpRawFU <- trunc(median(tmpRawFU))

}

## return v-scale score for raw scores

study$v_bl[i] <- useLookups[which(study$agebl[i] >= useLookups$MinMonths_GE &

study$agebl[i] < useLookups$MaxMonths_LT &

tmpRawBL >= useLookups$minscore &

tmpRawBL <= useLookups$maxscore),"vscale"]

study$v_fu[i] <- useLookups[which(study$agefu[i] >= useLookups$MinMonths_GE &

study$agefu[i] < useLookups$MaxMonths_LT &

tmpRawFU >= useLookups$minscore &

tmpRawFU <= useLookups$maxscore),"vscale"]

}

## ANCOVA for treatment effect on GSV

gsv.lm <- lm(gsv_fu_obs ~ gsv_bl_obs + group, data = study)

v.lm <- lm(v_fu ~ v_bl + group, data = study)

## Return dataframe with estimated mean difference, se of difference, and p

## Sometimes an error occurs due to singularity (lack of variability in V-scale), produces NAs except for sig columns which are set to false to indicate failure to detect effect

output <- data.frame(scenario=scenario,

pV = ifelse(length(summary(v.lm)$coefficients) < 12,

NA_real_, summary(v.lm)$coefficients[3,4]),

pGSV = ifelse(length(summary(gsv.lm)$coefficients) < 12,

NA_real_, summary(gsv.lm)$coefficients[3,4]),

sigV= ifelse(length(summary(v.lm)$coefficients) < 12,

FALSE, summary(v.lm)$coefficients[3,4] < .05),

sigGSV= ifelse(length(summary(gsv.lm)$coefficients) < 12,

FALSE, summary(gsv.lm)$coefficients[3,4] < .05),

estV = ifelse(length(summary(v.lm)$coefficients) < 12,

NA_real_, summary(v.lm)$coefficients[3,1]),

estGSV = ifelse(length(summary(gsv.lm)$coefficients) < 12,

NA_real_, summary(gsv.lm)$coefficients[3,1]),

seV = ifelse(length(summary(v.lm)$coefficients) < 12,

NA_real_, summary(v.lm)$coefficients[3,2]),

seGSV = ifelse(length(summary(gsv.lm)$coefficients) < 12,

NA_real_, summary(gsv.lm)$coefficients[3,2]))

return(output)

}

## ---------------------------------------------------------------------------------------------

## conversions()

## ---------------------------------------------------------------------------------------------

## Convert results of simFX to a semi-flattened list

##

## Arguments:

## x - object outputted by simFX

##

## Return values:

## List which has been partially flattened

## ---------------------------------------------------------------------------------------------

conversions <- function(x){

temp <- matrix(unlist(x), ncol=9, byrow=T)

temp <- as.data.frame(temp)

colnames(temp) <- c("scenario","pV","pGSV","sigV","sigGSV","estV","estGSV","seV","seGSV")

return(temp)

}

# Run simulation

library(pbapply)

set.seed(53243)

startTime <- proc.time()

simresults <- list()

for(i in 1:nrow(combos)){

n <- ifelse(combos[i,]$es_size == 0, 10000, 5000) #10k reps for typeI, 5k for power

simresults[[i]] <- pbreplicate(n,simFX(i))

}

warnings()

endTime <- proc.time()

endTime - startTime

saveRDS(simresults, "../tables/simsOut_02_25_2022.RDS")

# Summarize results of simulation

## Interested primarily in power (type I error where es = 0)

tempsum <- bind_rows(lapply(simresults, conversions)) %>%

left_join(combos, by = "scenario")

simsummary <- bind_rows(lapply(simresults, conversions)) %>%

left_join(combos, by = "scenario") %>%

mutate(sigV = case_when(is.nan(pV) ~ 0,

T ~ sigV),

dV = estV / (seV*sqrt(66)),

dGSV = estGSV / (seGSV*sqrt(66))) %>%

group_by(scenario) %>%

summarize(nValid_v = length(which(!is.na(pV))),

nValid_gsv = length(which(!is.na(pGSV))), #nvalid < 5k or 10k indicates floor effect error

power_v = sum(sigV) / length(sigV),

power_gsv = sum(sigGSV) / length(sigGSV),

medd_v = median(dV, na.rm=T), # floor effects removed from calculation

medd_gsv = median(dGSV, na.rm=T),

iqrd_v = IQR(dV, na.rm = T),

iqrd_gsv = IQR(dGSV,na.rm=T),

meddiff_v = median(estV, na.rm=T),

meddiff_gsv = median(estGSV, na.rm=T)) %>%

ungroup()
